# A Novel Splice-Site Mutation in Angiotensin I-Converting Enzyme (ACE) Gene, c.3691+1G>A (IVS25+1G>A), Causes a Dramatic Increase in Circulating ACE through Deletion of the Transmembrane Anchor

**DOI:** 10.1371/journal.pone.0059537

**Published:** 2013-04-01

**Authors:** Alexandre Persu, Michel Lambert, Jaap Deinum, Marta Cossu, Nathalie de Visscher, Leonid Irenge, Jerôme Ambroise, Jean-Marc Minon, Andrew B. Nesterovitch, Alexander Churbanov, Isolda A. Popova, Sergei M. Danilov, A. H. Jan Danser, Jean-Luc Gala

**Affiliations:** 1 Pole of Cardiovascular Research, Institut de Recherche Expérimentale et Clinique, Université Catholique de Louvain, Brussels, Belgium; 2 Division of Cardiology, Cliniques Universitaires Saint-Luc, Université Catholique de Louvain, Brussels, Belgium; 3 Division of Internal Medicine, Cliniques Universitaires Saint-Luc, Université catholique de Louvain, Brussels, Belgium; 4 Department of Internal Medicine, Radboud University Medical Center, Nijmegen, The Netherlands; 5 Department of Rheumatology, Radboud University Medical Center, Nijmegen, The Netherlands; 6 Department of Rheumatology, University Medical Center, Utrecht, The Netherlands; 7 Center of Applied Molecular Technologies, Institut de Recherche Expérimentale et Clinique, Université catholique de Louvain, Brussels, Belgium; 8 Department of Laboratory Medicine, Transfusion and Thrombosis-Haemostasis Unit, CHR de la Citadelle, Liège, Belgium; 9 Department of Dermatology, Rush University, Chicago, Illinois, United States of America; 10 Biology Department, New Mexico State University, Las Cruces, New Mexico, United States of America; 11 Institute for Chemistry of Life, Northwestern University, Evanston, Illinois, United States of America; 12 Institute for Personalized Respiratory Medicine, University of Illinois at Chicago, Chicago, Illinois, United States of America; 13 Department of Anesthesiology, University of Illinois at Chicago, Chicago, Illinois, United States of America; 14 Division of Pharmacology and Vascular Medicine, Department of Internal Medicine, Erasmus University, Rotterdam, The Netherlands; Max-Delbrück Center for Molecular Medicine (MDC), Germany

## Abstract

**Background:**

Angiotensin-converting enzyme (ACE) (EC 4.15.1) metabolizes many biologically active peptides and plays a key role in blood pressure regulation and vascular remodeling. Elevated ACE levels are associated with different cardiovascular and respiratory diseases.

**Methods and Results:**

Two Belgian families with a 8-16-fold increase in blood ACE level were incidentally identified. A novel heterozygous splice site mutation of intron 25 **-** IVS25+1G>A (c.3691+1G>A) **-** cosegregating with elevated plasma ACE was identified in both pedigrees. Messenger RNA analysis revealed that the mutation led to the retention of intron 25 and Premature Termination Codon generation. Subjects harboring the mutation were mostly normotensive, had no left ventricular hypertrophy or cardiovascular disease. The levels of renin-angiotensin-aldosterone system components in the mutated cases and wild-type controls were similar, both at baseline and after 50 mg captopril. Compared with non-affected members, quantification of ACE surface expression and shedding using flow cytometry assay of dendritic cells derived from peripheral blood monocytes of affected members, demonstrated a 50% decrease and 3-fold increase, respectively. Together with a dramatic increase in circulating ACE levels, these findings argue in favor of deletion of transmembrane anchor, leading to direct secretion of ACE out of cells.

**Conclusions:**

We describe a novel mutation of the ACE gene associated with a major familial elevation of circulating ACE, without evidence of activation of the renin-angiotensin system, target organ damage or cardiovascular complications. These data are consistent with the hypothesis that membrane-bound ACE, rather than circulating ACE, is responsible for Angiotensin II generation and its cardiovascular consequences.

## Introduction

Angiotensin I-converting enzyme (ACE) is a Zn^2+^ carboxydipeptidase enzyme which transforms angiotensin I in angiotensin II, a potent vasoconstrictor, as well as other biologically active peptides. Through this metabolic process, ACE plays a key role in the regulation of blood pressure and also in the development of vascular pathology and remodeling [1]–[3]. In man, the gene coding ACE is mapped on chromosome 17 (17q23). It is 21 kilobases long and comprises 26 exons and 25 introns [4].

There are two different isoforms of ACE: somatic ACE, which is expressed in somatic tissues and testicular ACE, which is restricted to testes and germinal tissue [5] and generated due to alternative splicing [4], [6]–[7]. ACE is constitutively expressed on the surface of endothelial cells, different absorptive epithelial and neuroepithelial cells [8]–, and cells of the immune system -macrophages and dendritic cells [14]–[15]. Somatic ACE mRNA includes all 26 exons except exon 13 which is spliced, whereas testicular ACE mRNA includes exons 13 to 26 [4]. Somatic original ACE protein is constituted of 1306 aminoacids, including the peptide signal (position 1 to 29) [6], which is removed upon insertion of the enzyme in the plasma membrane. Both isoforms are plasma membrane-bound through a transmembrane domain spanning from valine 1228 to serine 1248, these positions being defined according to mature somatic ACE, after removal of peptide signal [16].

Serum ACE originates likely from endothelial cells [17] through proteolytic cleavage by an unknown secretase [11], [17]–[21]. The release of soluble ACE in plasma is achieved through cleavage of Arg1203-Ser1204 peptide bond in the stalk region of ACE, near the transmembrane segment [20].

In healthy individuals, the circulating ACE level is very stable [22], as opposed to higher concentrations of circulating ACE in patients with granulomatous diseases (sarcoidosis in particular) and Gaucher’s disease [23]–[25]. During the past decade, different mutations in somatic ACE have been reported to associate with high serum ACE levels [26]–[28].

We report here the identification of a novel splice site mutation of intron 25 (IVS25+1G>A) of the ACE gene, associated to a dramatic increase in levels of circulating ACE, without any apparent harmful effect on human health.

## Materials and Methods

### Ethics Statement

The study protocol was approved by the Faculty of Medicine and Health Sciences Research Ethics Committee of the Université catholique de Louvain (Belgium) and the procedures to be followed were in accordance with institutional guidelines. Adult subjects gave written informed consent for DNA extraction, blood analysis and the captopril test. For the single minor participant, written informed consent was also obtained from both parents. The consent procedure was approved by the Ethics Committee.

### Study Participants

A dramatic increase in circulating ACE level was detected in a 41- year-old Belgian normotensive lady during a thorough work-up for fatigue. No evidence of sarcoidosis or of other granulomatous diseases was found. Consequently, circulating ACE level and ACE genotype determination were assessed for the patient, her daughter and 7 relatives, as well as her husband (used as a control). Aside of ACE blood tests and genetic testing, a standardized medical work-up was proposed to all subjects enrolled in this study, including a detailed interview of past medical history and current complaints, blood pressure and heart rate measurements, as well as a determination of circulating components of the renin-angiotensin-aldosterone system (RAAS), both at baseline and 3 and 6 hours after oral intake of 50 mg captopril. Blood pressure was measured seated and after three minutes rest using a validated oscillometric device (OMRON M10-IT®). At each step, 3 measures were recorded at 1-minute-intervals. Baseline levels of serum creatinine and potassium, and urine protein-to-creatinine ratio were also determined.

Afterwards, a major elevation of plasma ACE, related to the same mutation, was also found in another apparently unrelated Belgian patient. Plasma ACE level was measured and genetic analysis of *ACE* was also performed in the index patient and available relatives, and relevant clinical information was obtained.

### Measurement of RAAS Parameters

In the first pedigree, circulating ACE level was measured using a colorimetric kit (Fujirebio Inc, Tokyo, Japan), with a reference range of 8.0–26 IU/L [29]. In the second pedigree, circulating ACE was measured using the ACE kinetic Kit (Bühlmann, Schönenbuch, Switzerland), with a reference range of 12–68 IU/L. Alternatively a fluorimetric assay [30]–[31] was used. Briefly, 20–40 µl aliquots of serum or plasma, diluted 1/5–1/50 in PBS-BSA (0.1 mg/ml), were added to 200 µl of ACE substrate (5 mM Hip-His-Leu or 2 mM Z-Phe-His-Leu), and incubated for the appropriate time at 37°C. The His-Leu product was quantified by incubation with O-phthaldialdehyde spectrofluorometrically at 365 nm excitation and 500 nm emission wavelengths.

Total renin concentration was measured with a commercial immunoradiometric kit (Renin III, Cisbio, Gif-Sur-Yvette, France) [32]. The measurement was performed before and after the induction of a conformational change in the prorenin molecule with renin inhibitor aliskiren (10 µmol/L for 48 hours at 4°C), enabling renin recognition by active site-directed radiolabeled antibodies of the Cisbio kit [32].http://circ.ahajournals.org/cgi/content/full/117/25/3199 - R18-189866#R18-189866 Substraction of the renin concentration from the total renin concentration yielded the prorenin concentration. Angiotensinogen was measured as the maximum quantity of angiotensin (Ang) I that was generated during incubation with excess recombinant renin. Ang I and II were measured with sensitive radioimmunoassay [33]. Aldosterone was measured by solid-phase radioimmunoassay [32].

### Flow Cytometry of Dendritic Cells with Anti-ACE mAbs and ACE Shedding

Immature dendritic cells (DC) were generated from 2 individuals with elevated ACE and 2 non-affected family members as described before [26]. Briefly, peripheral mononuclear cells from the blood of affected and non-affected individuals were cultured for 6 days in conditions which generated their differentiation into immature dendritic cells. After 6 days, cells were harvested, washed and counted and media used for culture collected. Flow cytometric analysis was performed with 1 million of DC using 10 µg/ml of the mixture of anti-ACE primary mAbs - i2H5, 9B9 [15] and FITC-conjugated GAM IgG F(ab′)2 (Zymed, San Francisco, CA) as the secondary antibody on a FACScan flow cytometer (Becton and Dickinson & Co, Mountain View, CA). An isotype-matched mAb to unrelated antigen was used as a negative control. The remaining cells were lysed, and ACE activity was measured in the collected medium and the cell lysates using the Fujirebio kit (Fujirebio Inc, Tokyo, Japan).

### Immunological Characterization of the ACE Mutant (Plate Immunoprecipitation Assay)

Plate immunoprecipitation assay was carried out as described [34]–[35]. Briefly, 96-well plates (Corning, NY, USA) were coated with anti-ACE mAbs via goat anti-mouse IgG (Pierce, Rockford, IL) bridge and incubated for 3 hours with plasma samples. A panel of mAbs directed against 16 different epitopes located on the N- and C-domain of human ACE (‘conformational fingerprinting’) was used. Binding of several of these anti-ACE mAbs to circulating ACE is greatly influenced by dilution of the plasma/serum, because such dilution disturbs the equilibrium between ACE and ACE-binding protein(s) in blood [Danilov 2012, unpublished observations]. We therefore used as a comparator plasma samples from individuals with equally elevated ACE levels (to allow identical 1∶50 dilutions), i.e., from patients with a truncation of the C-terminal end of soluble ACE (W1197Stop, resulting in 1196 instead of the normal 1203 residues) [27], and from patients with a mutation (Y456D) that also causes familial elevation of blood ACE, but without a change in the C-terminal end of soluble ACE [28]. ACE activity, precipitated from the blood to the bottom of the wells by different mAbs, was measured by adding a substrate for ACE (His-His-Leu) directly into the wells [34]–[35].

### ACE Purification from Heparinized Plasma

ACE was isolated from plasma of 2 individuals with wild-type ACE and 2 subjects harboring the IVS25+G>A mutation, using affinity chromatography on a lisinopril-Sepharose column [36]. Briefly, plasma (4 mL) diluted 1∶2 in HEPES (20 mmol/L, pH 7.5, NaCl 150 mmol/L) was incubated with 5 mL of lisinopril-Sepharose and after intensive washing of unbound proteins, ACE was eluted with 50 mmol/L borate buffer, pH 9.5.

### Western Blot Analysis of Mutant ACEs

All samples for SDS electrophoresis were equilibrated to a final ACE activity 5 mU/mL (Hip-His-Leu as a substrate) and were run using gradient 4–15% Tris-HCl pre-cast SDS PAGE gels (Bio-Rad Laboratories, Hercules, CA). After electrophoretic transfer of proteins to microporous Immobilon-P PVDF membranes, each membrane was incubated in 10 mmol/L Tris-HCl (pH 8.0) buffer containing 150 mmol/L NaCl, 0.05% Tween 20, and 5% dry milk, prior to incubation with mAbs to denatured human ACE - 3C5 [37] and 5C8 [38] at a concentration of 2 µg/mL overnight at 4°C. Subsequent steps were carried out with goat-anti-mouse IgG conjugated with peroxidase (Jackson ImmunoResearch Laboratories, West Grove, PA), and peroxidase activity was developed using Amersham ECL Western Blotting Detection Reagent (GE Healthcare, UK).

### Genomic DNA Sequencing and Genotyping of the ACE Gene

#### DNA and RNA extraction

Peripheral blood mononuclear cells (PBMC) were isolated from whole blood of the siblings as well as from the controls, using Ficoll Hypaque®. DNA was extracted using the QIAamp® DNA Blood Mini Kit (Qiagen, Hilden, Germany) according to the manufacturer’s protocol. The extracted DNA was stored at −20°C until use. Total RNA was isolated from PBMC with TRIzol® reagent (Invitrogen Life, Merelbeke, Belgium) according to the manufacturer’s protocol and stored at −80°C until use.

#### DNA analysis

For each participant to the study, ACE gene was screened for best known mutations by PCR amplification and sequencing. In practice, the gene was screened for the insertion/deletion of the Alu repeats within intron16 [39], the nonsense 1197^th^ codon TGG→TGA mutation [27] and the missense 1199^th^ codon CCG→CTG mutation associated with the Pro1199Leu substitution using primers as described [26].

This step was followed by amplification and subsequent sequence analysis using primers pairs encompassing all the ACE open reading frames (ORFs) and the exon-intron boundaries for additional mutations (Table 1). The primers were designed against the reference ACE gene sequence (AY436326) using the Primer3 software and purchased from Eurogentec (Seraing, Belgium). Taq Gold DNA polymerase (Applied Biosystems, Nieuwekerke, The Netherlands) was used for PCR amplification of 250 ng of genomic DNA, using the following reaction mixture : 5 µL of PCR buffer (10 mM Tris hydrochloride, pH 8.3 and 50 mM potassium hydrochloride), 1.5 mM of MgCl2, 200 µM of each deoxyribonucleoside triphosphate (dNTPs), 0.2 µM of each primer in a final volume of 50 µL. Cycling conditions were as follows: 5 min at 95°C; 30 cycles of 95°C for 40 s, 60°C for 40 s, and 72°C for 1 min 20 s; and final extension of 7 min at 72°C. The amplification was carried out in a Gene Amp PCR System 2700 (Applied biosystems). The resulting PCR products were visualized on a 2% ethidium-bromide stained agarose gel. The amplicons were then purified with Microcon (Amicon, Millipore Corporation, Bedford, MA 01730 USA) columns and sequenced in both orientations on an automated ABI 3130 capillary sequencer (PRISM; Applied Biosystems, Nieuwerkerk, the Netherlands), using the Big Dye Deoxy Terminator Cycle Sequencing kit from the same manufacturer.

**Table 1:** Primers used for PCR amplification of the exons and exon-intron boundaries of the ACE gene (accession number AY436326).

Name	Sequence (5′->3′)	Position	Expected amplicon size (bp)
ACE1-FOR	GGGGGTGTGTCGGGTT	2296–2311	253
ACE1-REV	TCGGCGCTGGAGTTGTA	2532–2548	
ACE2-FOR	CCTTTATGGCCTGCATCTA	3151–3170	1285
ACE2-REV	GGGCAATGTGGGAACACT	4417–4435	
ACE3-FOR	GTGAAGGCCGTTGAAGACT	4986–5004	865
ACE3-REV	ACTTCCGTGGGACTCATG	5832–5850	
ACE4-FOR	ACTCAGCGATGCATGAAGAA	6301–6320	743
ACE4-REV	AGAACGTGCTCTTCCCATGT	7026–7045	
ACE5-FOR	TGTCTTTCAGGTGCCAATC	7658–7676	332
ACE5-REV	CCTCTCAGCCCTCCCATA	7972–7989	
ACE6-FOR	TTCCCTCTCCTTTCATCTCA	8279–8298	1060
ACE6-REV	CATCAGAGCAGCCAGACA	9321–9338	
ACE7-FOR	GCCCATGGTACCCACTCT	9584–9601	1131
ACE7-REV	GGGACAGCATCTCTGTGTGT	10696–10715	
ACE8-FOR	AGCCCTCAGCTCCCACTT	11753–11770	632
ACE8-REV	CTCACTCAGCCTGCACAT	12366–12384	
ACE9-FOR	CAAGCAGAGGTGAGCTAAGG	13878–13897	585
ACE9-REV	CACTGTGGTCCGTCTTTACC	14442–14461	
ACE10-FOR	GAGGGCATTGAGCCTAAGTA	16171–16190	562
ACE10-REV	GACCCTTTGCTTGGTTCAT	16715–16733	
ACE11-FOR	CACTGTCCTCTCCCAACAC	18672–18690	1135
ACE11-REV	ACACAAAGCTGTGGGTACTG	19787–19806	
ACE12-FOR	AAGGCCCTCAACCAACTC	21626–21643	680
ACE12-REV	GTTAAGACCCAAGGCTGGAG	22286–22305	
ACE13-FOR	CATGCTGTCTCCTTGCTT	22391–22408	262
ACE13-REV	GTCACCTCAGGAGTGTCTCA	22634–22653	

Detection of the IVS25+1G>A mutation using Restriction Length Fragment Polymorphism Analysis is described in the Data S1 and Table S1.

#### Transcript analysis

In order to perform ACE transcript analysis, 1.0 µg of total RNA extracted from PBMC of the 16 people from the two unrelated families and from controls were digested with 1.U of DNAse I (Invitrogen, Merelbeke, Belgium) in 10 µL of a mix containing 20 mmol/L Tris-HCl, pH 8.4, 2 mmol/L MgCl_2_ and 1 U/µL of RNase-Inhibitor (Roche, Boehringer Mannheim, Germany) during 15 min at 25°C. The reaction was stopped by adding 1 µL of EDTA 25 mmoles/L. After denaturation at 70°C during 10 min, 1.0 µg of ice-chilled total RNA was reverse-transcribed with 1 µL of random hexamers (0.7 *µ*mol/L; Eurogentec; Belgium), 4 U/µL of Superscript™ RNase H^−^ reverse transcriptase (Invitrogen, Merelbeke, Belgium), 0.35 mmol/L dNTPs, 50 mmol/L Tris-HCl, pH 8.3, 75 mmol/L KCl, 3 mmol/L MgCl_2_ and 100 mmol/L dithiothreitol, in a final volume of 50 µL. The cDNA synthesis was performed at 42°C for 1 h.

PCR amplification of the cDNA from 24^th^ to 26^th^ intron was carried out on 2 µL RT product in a final volume of 50 µL, using primers ACE2425-F and ACE2425-R (Table 2). The amplification, visualization and subsequent sequence analysis were carried out as described above.

**Table 2:** Primers used for PCR amplification of the ACE cDNA (NM_000789.3) region encompassing the IVS25 splice-junction site.

Name	Sequence (5′->3′)	Position	Expected amplicon size (bp)
ACE2425-FOR	GTGCCTTACATCAGGTACTTTGT	3401–3423	561
ACE2425-REV	GTCACCTCAGGAGTGTCTCA	3942–3961	

### *In silico* Prediction Analysis

Bioinformatics analysis of potential splicing aberration was done using different Web-based programs designed to detect putative splice sites, taking into account branch points, exonic and intronic motifs, and several regulatory proteins.

- Splice Scan II (http:/www.splicescan2.lumc.edu) [40].
- CRYP-SKIP (http:/www.dbass.org.uk/cryp-skip) [41].

## Results

### Clinical Data

#### First pedigree

The index patient, a 41-year-old Caucasian female was referred in November 2006 to the Internal Medicine Department of the Cliniques Universitaires Saint-Luc because of fatigue and headache persisting for several months, as well as of incidentally found plasma ACE level far above the normal range (492 IU/L with the ACE kinetic kit, normal range: 8–52 IU/L). The patient had neither relevant past medical history nor other complaints and did not take any medication. Blood pressure on admission was 110/70 mmHg. Besides few infracentimetric cervical lymphadenopathies, clinical examination was unremarkable. Determination of ACE activity after another blood sampling from this index patient with ACE colorimetric kit confirmed the high level of circulating ACE (146 IU/L), more than five times the upper limit of the assay (normal range 8–26 IU/L). Electrocardiogram and echocardiography were normal and, in particular, there was no evidence of left ventricular hypertrophy. A thorough work-up, including detailed blood and urine analysis and total body FDG-PET scan allowed to rule out sarcoidosis or other granulomatous diseases. By the end of the work-up, the symptoms had improved spontaneously.

**Figure 1:**
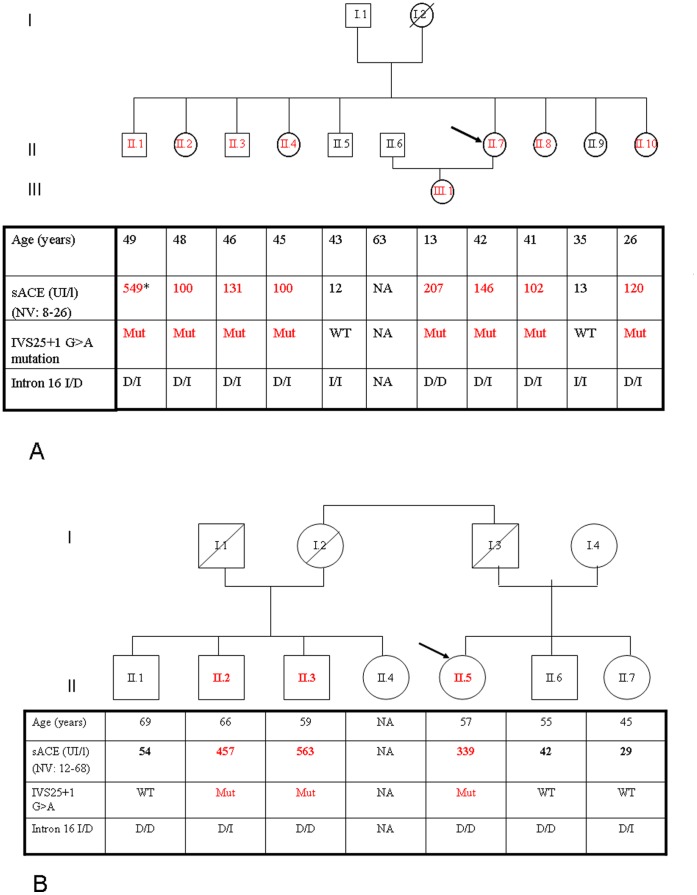
Genealogic tree of the two families harboring the IVS25+1G>A mutation. Genealogic tree of the two Belgian families harboring the IVS25+1G>A mutation, showing the circulating ACE levels and the genotypes at the IVS25+1 and I/D loci. Circulating ACE was determined with the Fujirebio kit in the individuals of the first pedigree (**1A**) except II.1, and with the ACE kinetic kit in individual II.1 of the first pedigree and in all individuals of the second pedigree (**1B**). This explains the higher values in individual II.1 compared to his siblings in the first pedigree. Arrow: index patient. NA: not analyzed; WT: wild type; Mut: presence of the IVS25+1 G>A mutation at the heterozygous state; I, insertion; D, deletion.

Due to very high elevation of blood ACE level (much higher that in the case of sarcoidosis or Gaucher diseases [23], [24], comparable with blood ACE levels characteristic for ACE mutations [26]–[28] the hypothesis of a familial elevation of circulating ACE level was considered. ACE levels were determined in the siblings (median age: 44 years, range 26–49 years) and the 13 year-old daughter of the index patient. Circulating levels of ACE differ 3-fold in the normal population [22], [42]. Therefore, in order to estimate correctly the effect of the mutation on ACE levels, it is more appropriate to compare the level of blood ACE in a given patient to members of his/her family, than to upper level of normal range. A 8–16-fold increase in plasma ACE activity was found in 7 out of 9 tested subjects (Figure 1A). The hyper-ACE segregation pattern was compatible with autosomal dominant inheritance. All subjects with elevated circulating ACE were normotensive at the time of clinical assessment. However, three of the them (II.1, II.8 and II.10), aged 49, 41 and 26 year-old were taking an antihypertensive drug (a fixed combination of lisinopril 20 mg and hydrocholorothiazide 12.5 mg, bisoprolol 5 mg, and telmisartan 80 mg, respectively). The youngest (II.10) suffered from chronic kidney disease with stage 4 renal insufficiency probably favored by neonatal hypoxia and hypoplasia of the right kidney. The five other subjects harboring the mutation were normotensive without treatment and had no evidence of overt cardiovascular or renal disease. Electrocardiogram (n = 5) and echocardiography (n = 2) showed no evidence of left ventricular hypertrophy or other significant abnormality. Plasma potassium (mean: 3.9 mEq/l, range: 3.8–4.3 mg/dl) and, with the exception of II.10, plasma creatinine (mean: 0.84 mg/dl, range: 0.74–1.14 mg/dl), glomerular filtration assessed by the MDRD formula (mean: 86 range: 69–115 ml/min/1.73 m^2^), as well as urine protein-to-creatinine ratio (mean: 55 mg/g; range: 0–119 mg/g) were all within normal limits.

#### Second pedigree

In 2005, an otherwise asymptomatic 55-years old Caucasian female patient with a past history of Grave’s disease presented with a left facial hemispasm. Clinical examination was otherwise unremarkable. She was normotensive and had no left ventricular hypertrophy on echocardiography. A determination of plasma ACE level was asked in order to rule out a granulomatous disease. The latter was found to be dramatically increased (339 IU/L with ACE kinetic kit and normal range 12–68 IU/L). A comprehensive work-up including blood and urine analysis, CT-scan and Gallium-67 scintigraphy failed to show evidence of sarcoidosis, tuberculosis or another granulomatous disease. Subsequently, the patient was offered yearly thorax CT-scan which remained negative, whereas plasma ACE was consistently in the range of 330–360 IU/L.

Two of four paternal first-degree cousins of the index patient, aged 59 and 66 years, also had a marked elevation of circulating ACE (Figure 1B). Interestingly, the first cousin (II.6) had been treated with corticosteroids for sarcoidosis at the age of 46. The diagnosis was based on serendipitous finding of pulmonary interstitial pattern, associated with increased lymphocytosis at broncho-alveolar lavage and markedly elevated circulating ACE levels. No biopsy was performed. While interstitial lesions on chest X-ray and computed tomography were in favor of an advanced form of sarcoidosis, the patient had no respiratory symptoms and carbon monoxide diffusion was only minimally altered. Based on disappearance of the radiologic interstitial syndrome, the patient was considered as cured despite a persistent increase in circulating ACE levels. Of note, by that time (in 1999) the first publication describing a mutation in ACE causing elevation of blood ACE was not reported [26]. However two papers identifying familial elevation of blood ACE levels in Japan [43] and Italy [44] had already been published.

To the best of our knowledge, the two pedigrees are unrelated. While the father and mother of the first sibship came from Tournai (province of Hainaut, French-speaking southwest part of Belgium) and Deinze (province of East Flanders, Flemish northeast part of Belgium), respectively, the second pedigree was originally from Jalhay (Province of Liège, French-speaking southeast part of Belgium).

### Response to Captopril

**Figure 2:**
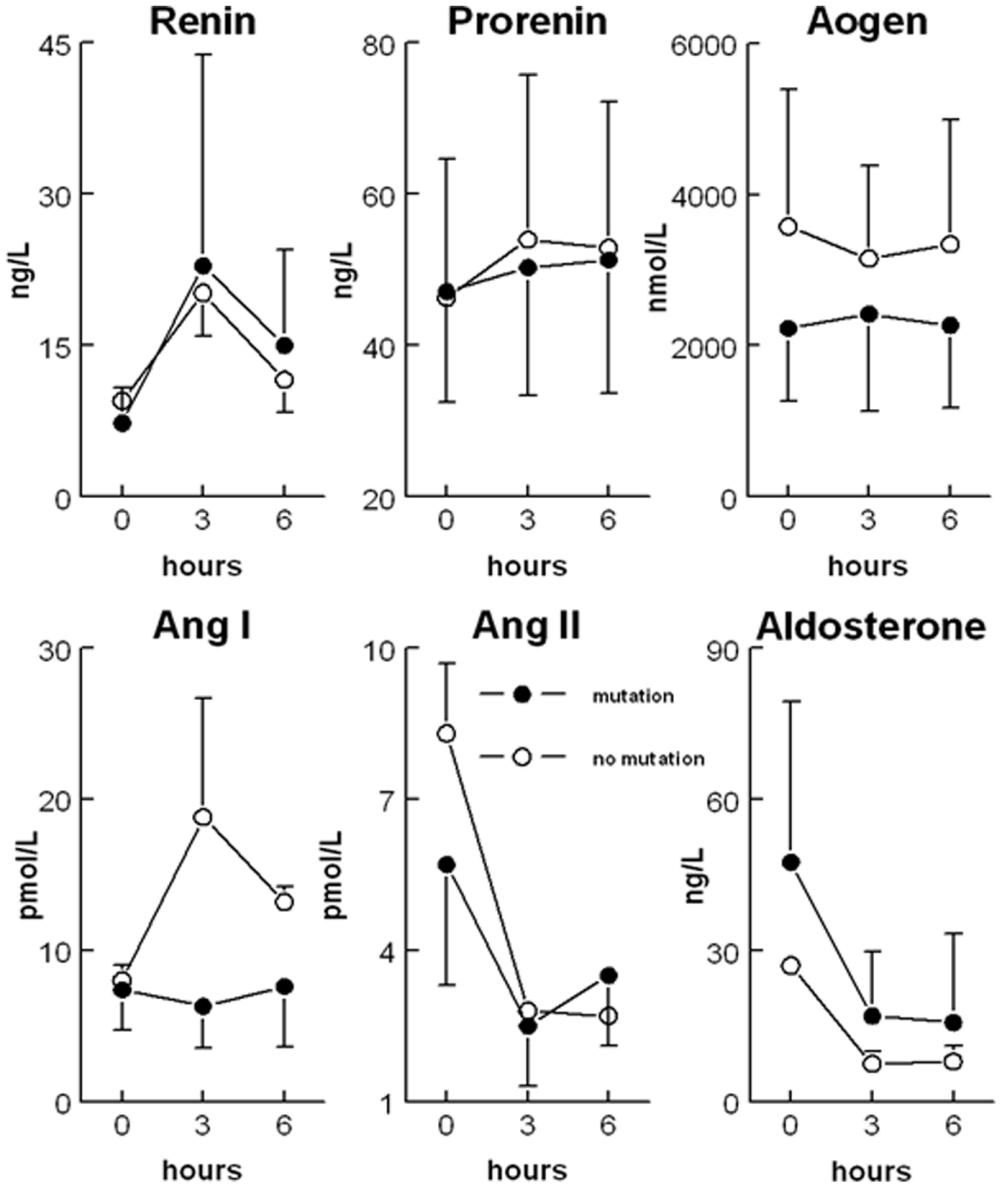
Plasma values of renin-angiotensin-aldosterone system components at baseline and after captopril. Plasma values of renin-angiotensin-aldosterone system components at baseline and after oral intake of 50 mg captopril at t = 0 in 6 affected and 2 unaffected family members. Aogen: angiotensinogen; Ang: angiotensin.

In the first pedigree, 50 mg captopril was given orally to the index patient, her daughter, her husband and 5 siblings. II.1 and II.10 were excluded because they already took RAS blockers. Baseline renin (7±4 *vs*. 9±1 ng/L), prorenin (47±16 *vs*. 46±18 ng/L), angiotensinogen (2232±963 *vs*. 3575±1812 nmol/L), Ang I (7±3 vs. 8±1 pmol/L), Ang II (6±2 *vs*. 8±1 nmol/L), and aldosterone (48±32 *vs*. 27±1 ng/L) levels were similar in affected (n = 6) and non-affected (n = 2) family members. Furthermore, with the exception of Ang I, the captopril-induced changes in RAAS components (2 to 3-fold rises in renin, 2 to 3-fold decreases in Ang II and aldosterone, and no change in prorenin or angiotensinogen) were identical in both groups (Figure 2). Ang I rose in parallel with renin in the non-affected family members only.

### Flow Cytometry of Dendritic Cells with Anti-ACE mAbs and ACE Shedding

**Figure 3:**
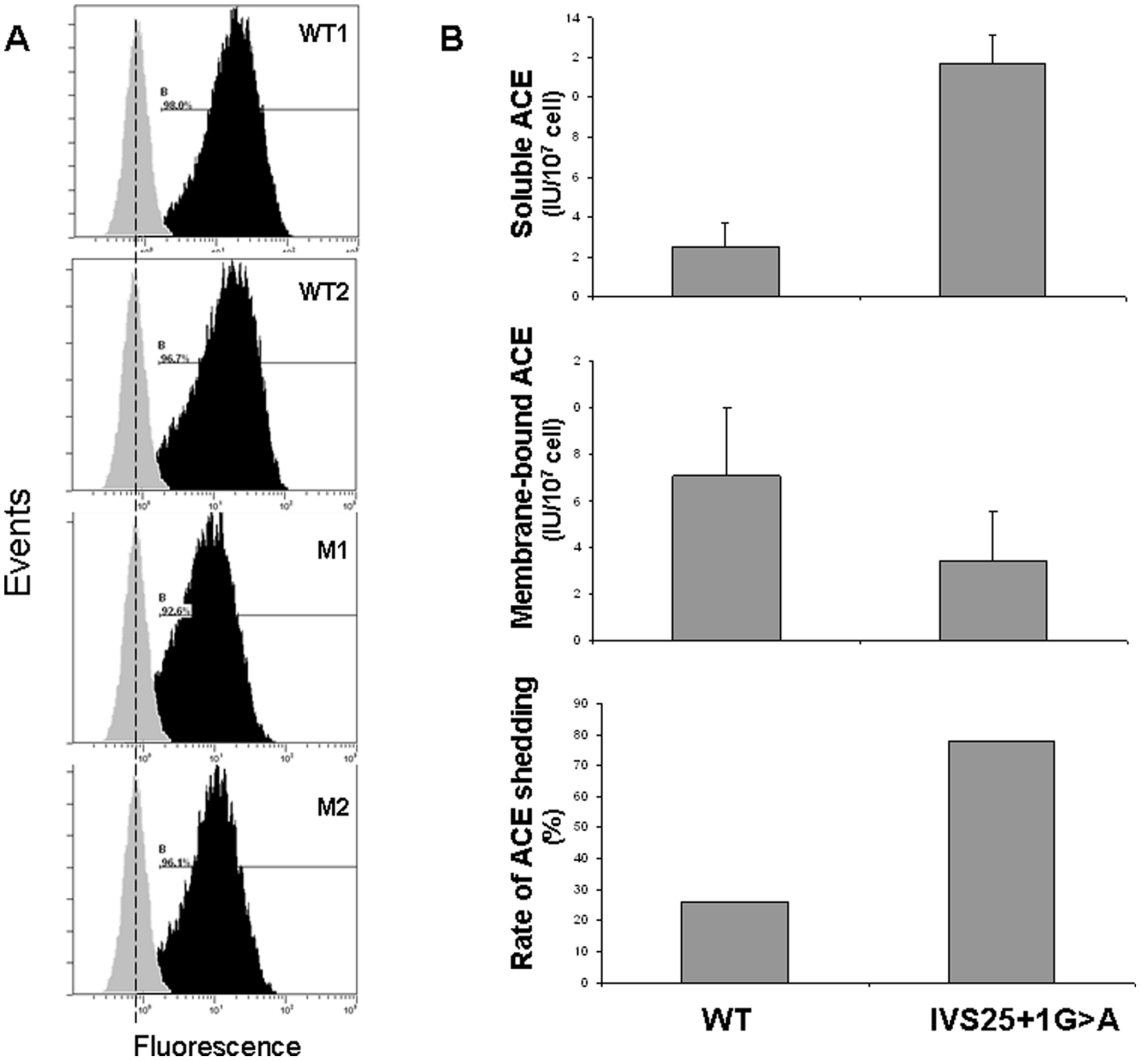
Cell surface expression and shedding of mutant ACE. **Panel A. FACS analysis of immature dendritic cells with anti-ACE antibodies.** Cells were stained with mAb i2H5 (black histogram) or with control mouse IgG (grey histogram). Figures correspond to values of median fluorescence intensity. Affected family members with the IVS25+1G>A mutation were shown to express a ≈50% lower level of ACE on their cell surface compared to non-affected family members. **Panel B. ACE shedding into the medium.** ACE activity in the medium (“Soluble ACE”) and membrane-bound ACE activity were determined on immature dendritic cells from 2 subjects harboring the IVS25+1G>A mutation (II.7 and III.1) and 2 controls (II.6 and I.9) from the first pedigree. Their ratio represents the rate of ACE shedding into the medium. In agreement with the findings of the FACS analysis, shedding was ≈3 times larger in the individuals with the mutated ACE. WT: wild-type subjects.

FACS analysis of dendritic cells demonstrated that affected family members with the IVS25+1G>A mutation expressed a ≈50% lower level of ACE on their cell surface versus non-affected family members (Figure 3A). In line with this observation, the cellular (membrane-bound) ACE activity levels in dendritic cells from affected family members were lower, while their medium (soluble) ACE levels were higher, which resulted in an at least 3-fold increase of ACE shedding in affected versus non-affected family members (Figure 3B).

### DNA Sequence Analysis

Genetic analysis of the ACE gene was performed in both families. While genetic screening for previously described mutations in ACE gene proved negative, a new heterozygous splice site mutation in the first nucleotide in GT dinucleotide-canonical 5′ donor splice site of intron 25 (IVS25+1G>A) was identified in both index patients and subsequently found to cosegregrate with elevated circulating ACE in all available relatives (Figure 1A and 1B). The intron 16 insertion/deletion (I/D) polymorphism [39] was present in both families. D/D, D/I and I/I genotypes were found in five, nine and two subjects, respectively and not assessable in two family members (Figure 1 A–B).

### Possible Mechanisms of Transmembrane Anchor Elimination

Mutations affecting human splice sites can lead to two major phenotypes, the exon skipping and activation of the cryptic splice sites of the same type located nearby of affected splice sites [41]. *In silico* analysis of the *ACE* gene (Data S1, Figures S1, S2, S3) demonstrates that the IVS25+1G>A (c.3691+1G>A) mutation can lead to three possible scenarios: i) skipping of 25^th^ exon; 2) full retention of 25^th^ intron; 3) generation of aberrant cryptic 5′donor site. In all three scenarios a Premature Termination Codon will be generated, leading to deletion of the transmembrane anchor.

### Immunological Characterization of the Mutant ACE

**Figure 4:**
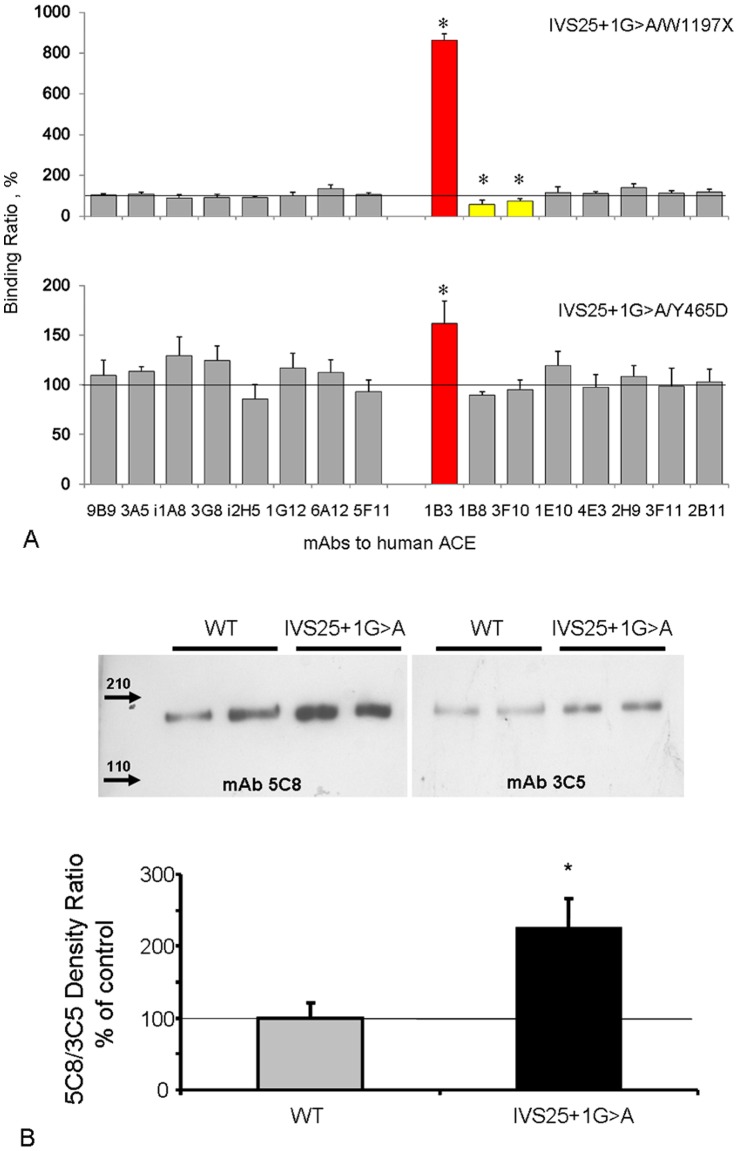
Analysis of the mutant ACE protein. **Panel A. Conformational fingerprinting of mutant ACEs with a set of mAbs to ACE.** Values were normalized versus individuals with the Trp1197Stop mutation or the Tyr465Asp mutation, since their blood contained similarly elevated ACE levels and allowed measurements at the same dilution. *P<0.05 vs. 100%. **Panel B.**, **Western blot analysis of wild-type (WT) and mutant ACEs.** Insert, 5C8/3C5 density ratio. *P<0.05 vs. WT.

A panel of mAbs directed against 16 different epitopes located on the N-and C-domain of catalytically active human ACE was used to characterize the conformation of ACE from affected family members (conformational fingerprinting’; Fig. 4A). Among these mAbs, 1B3 recognizes an epitope on the stalk region of ACE coded by exon 25 [38]. Its binding decreases in the case of a truncation of the C-terminal end of soluble ACE (W1197X) [27], but not in patients with a mutation (Y456D) that causes familial elevation of blood ACE without a change in the C-terminal end of soluble ACE [28]. Importantly, binding of this antibody to blood ACE from carriers of the IVS25+1G>A mutation was dramatically higher as compared to both W1197X and Y465D ACE, thereby ruling out scenario 1. In full agreement with this observation, binding of the mAbs 1B8 and 3F10 (which recognize the truncated ACE of W1179X carriers [27]) was lower in our affected family members versus W1197X ACE, but not versus Y465D ACE. These data indicate that the mutated ACE from the index patient still contains exon 25, and is immunologically different from previously described mutated ACEs.

Western blotting of ACE purified from the sera of 2 subjects carrying the IVS25+1G>A mutation and of two non-carriers, making use of mAb 3C5, which recognizes a sequential epitope at the beginning of the C-domain [45], and mAb 5C8, which recognizes an epitope at the C-terminal end of soluble ACE coded by exon 25 [27], [38], showed no size difference between soluble ACE from carriers and non-carriers (Figure 4B). However, the 5C8/3C5 ratio for ACE purified from sera of carriers (which will contain a mixture of mutant and wild-type ACE) was significantly higher than that of normal ACE. This further supports that skipping of exon 25 did not occur in carriers of the IVS25+1G>A mutation.

### mRNA and cDNA Analysis from Subjects Carrying IVS25+1G>A ACE Mutation

**Figure 5:**
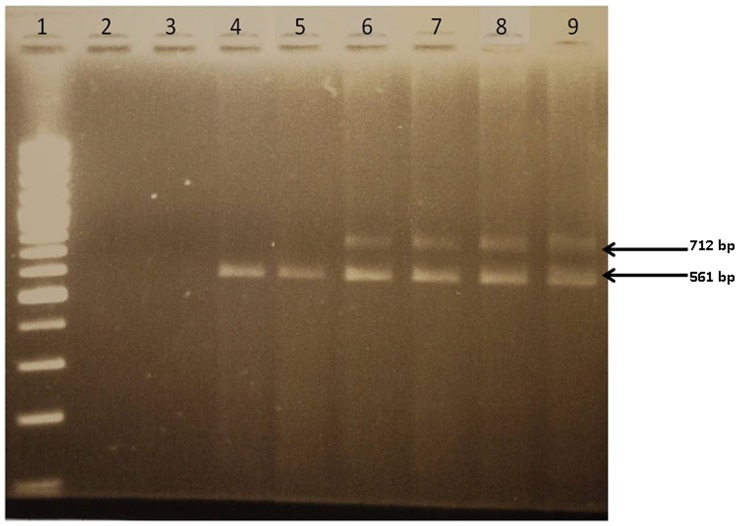
Analysis of cDNA from subjects harboring the IVS25+1G>A mutation versus controls. cDNA analysis of PCR products resulting from the amplification of cDNA from subjects harboring the IVS25+1G>A mutation versus wild type controls, visualised after electrophoresis on a 2% ethidium-bromide stained agarose gel. Ladder; Lanes 2–3: No Template Control (NTC); Lanes 4–5: two wild-type subjects for the IVS25+1G>A mutation; Lanes 6–9 : subjects heterozygous for the IVS25+1G>A mutation.

ACE cDNA analysis (after mRNA isolation and reverse transcription) from patients with the IVS25+1 G>A mutation in ACE gene provided definite evidence for the mechanism of transmembrane anchor elimination as a result of this mutation. PCR amplification of the regions flanking this mutation generated two ACE amplicons of 561-bp and 712-bp using cDNA from carriers of this mutation, whereas only the 561-bp amplicon was present in the normal subjects (Figure 5). Sequence analysis from both 561- and 712-bp amplicons revealed that the IVS 25+1 G>A mutation was present in the 712-bp fragment. The scheme presented in Figure S3 demonstrated that the appearance of the 712 amplicon could be only due to retention of 25^th^ intron. Therefore, we can state that this mutation resulted in the loss of the GT consensus splice donor site and a subsequent retention of 151-bp from intron 25 within the mRNA variant (accession number: provisional submission ID1529627), as predicted according to scenario 2. This frameshifted ACE mRNA splice variant leads to the appearance of a premature stop codon at position 3763, hence a premature termination of the protein.

## Discussion

Several molecular mechanisms underlying familial increases of circulating ACE have been described in the past decade. The Pro1199Leu substitution in the stalk region of ACE protein [26], [46] was found to be associated with a 5-fold increase in serum ACE activity in the blood of affected individuals from the Netherlands [26], Germany [47], and USA [48], in the absence of any clinical abnormality [26]. It has been suggested that this substitution leads to better accessibility of the cleavage site in the stalk region for ACE secretase, thus enhancing the cleavage secretion process [26], [46].

Then, a nonsense mutation at the codon for Trp1197 (TGG→TGA) identified in siblings was associated with a dramatic increase (13-fold) in circulating ACE level [27]. As a result, half the ACE expressed in these patients had a length of 1196 amino acids and lacked the transmembrane anchor, which starts from Val1228 [16]. Previous studies have shown that different recombinant ACE mutants also lacking the transmembrane anchor are not trafficked to the cell membrane but are directly secreted into the circulation [18], [49]. Interestingly, Y465D substitution in the N domain of ACE (far from the stalk region) was also associated with familial elevation of blood ACE. In this case the increase in ACE shedding depended on alterations in ACE dimerization [28].

We report here a novel splice site mutation of intron 25 **-** IVS25+1G>A - cosegregating with a 10-fold elevation of plasma ACE in two Belgian pedigrees (see Data S1, Table S2). As demonstrated by cDNA analysis, the latter leads to intron 25 retention, frameshift and premature stop codon upstream of the transmembrane domain of ACE.

The intron 25 **-** IVS25+1G>A variant is expected to yield a shorter ACE protein (1213 instead of 1306 amino acid residues) modeled at its C-terminal domain (see Data S1, Analysis in silico). This remodeling consists of the loss of the C-terminal 76 amino acids of the normal ACE protein and the addition of 12 novel aminoacids to the C-terminal protein end. Translation of this abnormal mRNA produces indeed a truncated ACE protein consisting of the Leu-1 to Ser-1201 expected amino acid stretch completed by a novel Asp-Thre-Ala-Thre-His-Pro-Thre-Ser-Ser-Leu-Gly-Ser stretch at its C-terminal end. Accordingly, the loss of the C-terminal domain harboring the Val-1228-to-Ser-1248 transmembrane domain likely leads to a direct shedding of the remodeled ACE into the circulation, hence contributing to the observed high level of circulating ACE in mutation carriers.

In agreement with these findings, in vitro analysis disclosed a 50% decrease in surface ACE expression in DC derived from patients harboring the mutant ACE, combined with a 3-fold increase in ACE shedding in comparison with non-affected members, and an altered “conformational fingerprinting” binding pattern of the mutated ACE to a panel of ACE antibodies.

As was the case for the Pro1199Leu substitution [26], this new mutation was associated neither with RAAS activation nor with a different responsiveness to ACE inhibition. Furthermore, no related cardiovascular damage could be demonstrated. These data are consistent with the hypothesis that membrane-bound ACE is exclusively responsible for Ang II generation and its cardiovascular consequences.

Of note, point mutations affecting the 5′donor splice-site, as identified in the current study, are rather common [50]. At this donor site, the most common mutations are those affecting the G residue at position +1, followed by mutations at position +5. Each of both donor site variants are thought to significantly reduce the pairing of the donor splice site with the complementary site in the small nuclear ribonucleoprotein particle U1snRNP, which is one of the first steps in the complex process of mRNA splicing [51]. Consequently, mutations at donor sites can either lead to exon skipping, as predicted in ten different IVS+1 mutations within the *RB1* gene and confirmed in vitro [50] or intron retention, as found in this work. A complex combination of insertion and deletion can also occur through activation of a cryptic splice site [50], [52].

In conclusion, comparative analysis of ACE protein and nucleic acids (genomic DNA, and cDNA) demonstrates unambiguously that the novel IVS25+1G>A splice site mutation is associated with retention of intron 25 within ACE mRNA. However, the normotensive status of carriers of this novel mutation shows evidence that high circulating serum ACE is not associated with hypertension. These data are consistent with the hypothesis that membrane-bound rather than secreted ACE is responsible for Ang II generation and its cardiovascular consequences. Indeed, transgenic mice expressing a form of ACE lacking the transmembrane domain (i.e., having soluble ACE only) are phenotypically similar to ACE-deficient mice [53].

Despite its absence of functional relevance or associated phenotype, the identification of a new mutation associated with a major increase of circulating ACE is of substantial clinical importance, as it will contribute to make a clear distinction between granulomatous diseases (namely sarcoidosis) and hyper-ACE-emia due to mutations (in this particular case, due to lack of transmembrane domain), thus preventing false diagnosis [47], unnecessary work-up [54] and long-term immunosuppressive treatment [47].

It should also be pointed out that the clinical features of the two index patients did not warrant ordering an ACE protein level. However, if analysis of human serum ACE is requested in the absence of any clear indication and if huge elevations are found as in the ACE mutations discovered so far, we would recommend considering a genetic analysis of ACE, rather than an extensive work-up directed at diseases that are known to increase ACE.

## Supporting Information

Figure S1 In silico analysis of the IVS25+1G>A mutation of ACE. **A**. A screenshot of the SpliceScan II output. Prediction for the wild type (exon 24-exon 26) contains the blue rectangle that schematically represents exon 25 with flanking 5′ (circle) and 3′ (triangle) splice sites. The X-axis indicates the location of the predicted exons along with splicing signals relative to the beginning of the fragment containing exon 25 surrounded by ±300nt intronic flanks. Mutation IVS25+1G>A converts the most significant dinucleotide GT to AT in the 5′ splice site thus removing the splice site out of the pool of available canonical 5′ splice sites flanking exon 25. Since none of the 5′splice sites predicted by the Bayesian splice sites sensor are located in the vicinity of the original 25^th^ exon 3′ flank, the predicted effect of the IVS25+1G>A mutation is exon skipping (no rectangle shown as result of mutation), i.e. there is no splicing-competent exon predicted in this region. **B**. A screenshot of the CRYP-SKIP output. Probability of cryptic splice site activation (P_CR-E_) found by the multivariate logistic discrimination procedure is shown on the right as a pointer balancing between exon skipping (EXSK) and cryptic splice site activation (CR-E). PCR-E is shown on the right as a pointer balancing between exon skipping (EXSK) and cryptic splicesite activation (CR-E). Values of each predictor variable used in the regression model are shown on the left where the last table row indicates PCR-E value of for the input sequence. Exon 25 sequence is in upper case and flanking introns 24 and 25 are in lower case. The program takes as input the wild type internal exon with some intronic flanks and evaluates chances of the same type cryptic splice sites activation located nearby of the original sites in case of a mutation eliminating the most significant GT/AG dinucleotides in the canonical 5′/3′ splice sites. Two predicted aberrant 5′ splice sites are shown as red arrows, with their corresponding normalized relative strength of 0.17 and 0.35. The blue triangle stands for the cryptic 3′ splice site that could potentially be activated in case of mutation affecting original 3′ splice site. The aberrant splice sites have been predicted by the NN Splice method (http://www.fruitfly.org/seq_tools/splice.html).
(TIF)

Figure S2 Premature Termination Codon Creation. Possible scenarios of the appearance of the Premature Termination Codon (PTC) as a result of IVS25+1G>A mutation in ACE. Fragment of the genomic DNA sequence. Exonic sequences were highlighted by yellow. Substitution of 1^st^ nucleotide in 25^th^ intron (G) by A shown by red color and underlined. **B.** PTC as a result of 25^th^ exon skipping. **C**. PTC as a result of full retention of 25^th^ intron. **D.** PTC as a result of aberrant 5′donor splice site and partial retention of 25^th^ intron. Part of the exonic sequence (25^th^ and 26^th^ exons) are in upper case and parts of the flanking intron (25^th^) are in lower case. Putative PTCs are highlighted in red. Non-ACE protein sequences are highlighted in blue.
(TIF)

Figure S3 Fragment of the genomic DNA sequence. Exonic sequences (24^th^–26^th^ exons) were highlighted by yellow. Substitution of 1^st^ nucleotide in 25^th^ intron (G) by A is shown by red color and underlined. Sequences of the primers that were used for amplification of intron-exon boundaries were highlighted with green. Amplification of cDNA (derived from mRNA) from subjects with wild type ACE (with normal level of blood ACE) gave band 561 bp. In the case of retention of 25^th^ intron an additional band of 712 bp appeared. Putative PTC was highlighted by red.
(TIF)

Table S1 Restriction Length Fragment Polymorphism Analysis of IVS25+1G>A mutation.
(DOC)

Table S2 Fold-change computation of ACE serum level according to genetic status of the patients.
(DOC)

Data S1 Includes *in silico* analysis of mutant ACE protein, RFLP identification of IVS25+1G>A ACE mutation and fold-change computation of ACE serum level according to genetic status of patients.
(DOC)
