# Supplementary figures and images for: A Novel Splice-Site Mutation in Angiotensin I-Converting Enzyme (ACE) Gene, c.3691+1G>A (IVS25+1G>A), Causes a Dramatic Increase in Circulating ACE through Deletion of the Transmembrane Anchor
Source: PLoS One. 2013 Apr 1;8(4):e59537. doi: 10.1371/journal.pone.0059537 (PMC3613373; doi:10.1371/journal.pone.0059537)

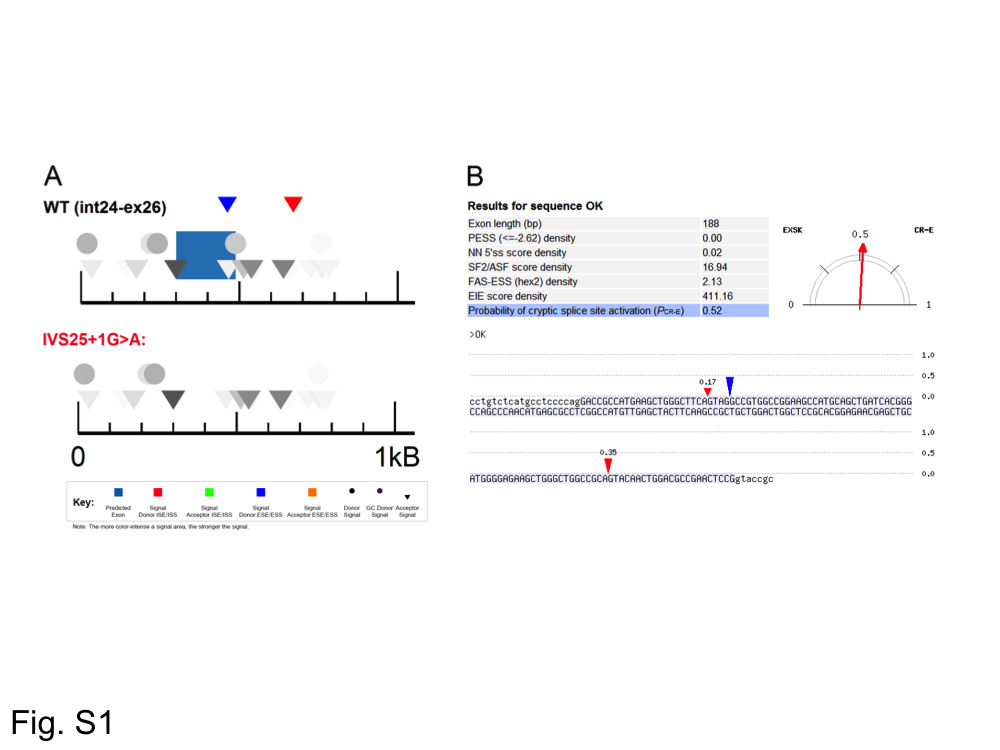

Supplement: Figure S1 — In silico analysis of the IVS25+1G>A mutation of ACE. A. A screenshot of the SpliceScan II output. Prediction for the wild type (exon 24-exon 26) contains the blue rectangle that schematically represents exon 25 with flanking 5′ (circle) and 3′ (triangle) splice sites. The X-axis indicates the location of the predicted exons along with splicing signals relative to the beginning of the fragment containing exon 25 surrounded by ±300nt intronic flanks. Mutation IVS25+1G>A converts the most significant dinucleotide GT to AT in the 5′ splice site thus removing the splice site out of the pool of available canonical 5′ splice sites flanking exon 25. Since none of the 5′splice sites predicted by the Bayesian splice sites sensor are located in the vicinity of the original 25th exon 3′ flank, the predicted effect of the IVS25+1G>A mutation is exon skipping (no rectangle shown as result of mutation), i.e. there is no splicing-competent exon predicted in this region. B. A screenshot of the CRYP-SKIP output. Probability of cryptic splice site activation (PCR-E) found by the multivariate logistic discrimination procedure is shown on the right as a pointer balancing between exon skipping (EXSK) and cryptic splice site activation (CR-E). PCR-E is shown on the right as a pointer balancing between exon skipping (EXSK) and cryptic splicesite activation (CR-E). Values of each predictor variable used in the regression model are shown on the left where the last table row indicates PCR-E value of for the input sequence. Exon 25 sequence is in upper case and flanking introns 24 and 25 are in lower case. The program takes as input the wild type internal exon with some intronic flanks and evaluates chances of the same type cryptic splice sites activation located nearby of the original sites in case of a mutation eliminating the most significant GT/AG dinucleotides in the canonical 5′/3′ splice sites. Two predicted aberrant 5′ splice sites are shown as red arrows, with their corresponding nor [file pone.0059537.s001.tif]

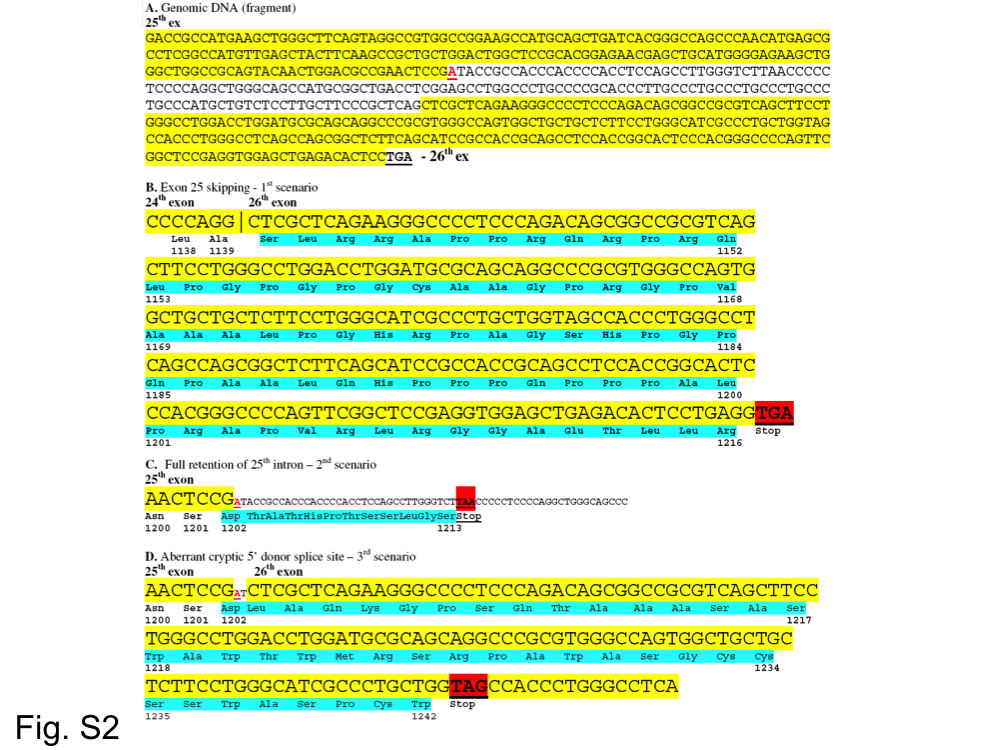

Supplement: Figure S2 — Premature Termination Codon Creation. Possible scenarios of the appearance of the Premature Termination Codon (PTC) as a result of IVS25+1G>A mutation in ACE. Fragment of the genomic DNA sequence. Exonic sequences were highlighted by yellow. Substitution of 1st nucleotide in 25th intron (G) by A shown by red color and underlined. B. PTC as a result of 25th exon skipping. C. PTC as a result of full retention of 25th intron. D. PTC as a result of aberrant 5′donor splice site and partial retention of 25th intron. Part of the exonic sequence (25th and 26th exons) are in upper case and parts of the flanking intron (25th) are in lower case. Putative PTCs are highlighted in red. Non-ACE protein sequences are highlighted in blue. (TIF) [file pone.0059537.s002.tif]

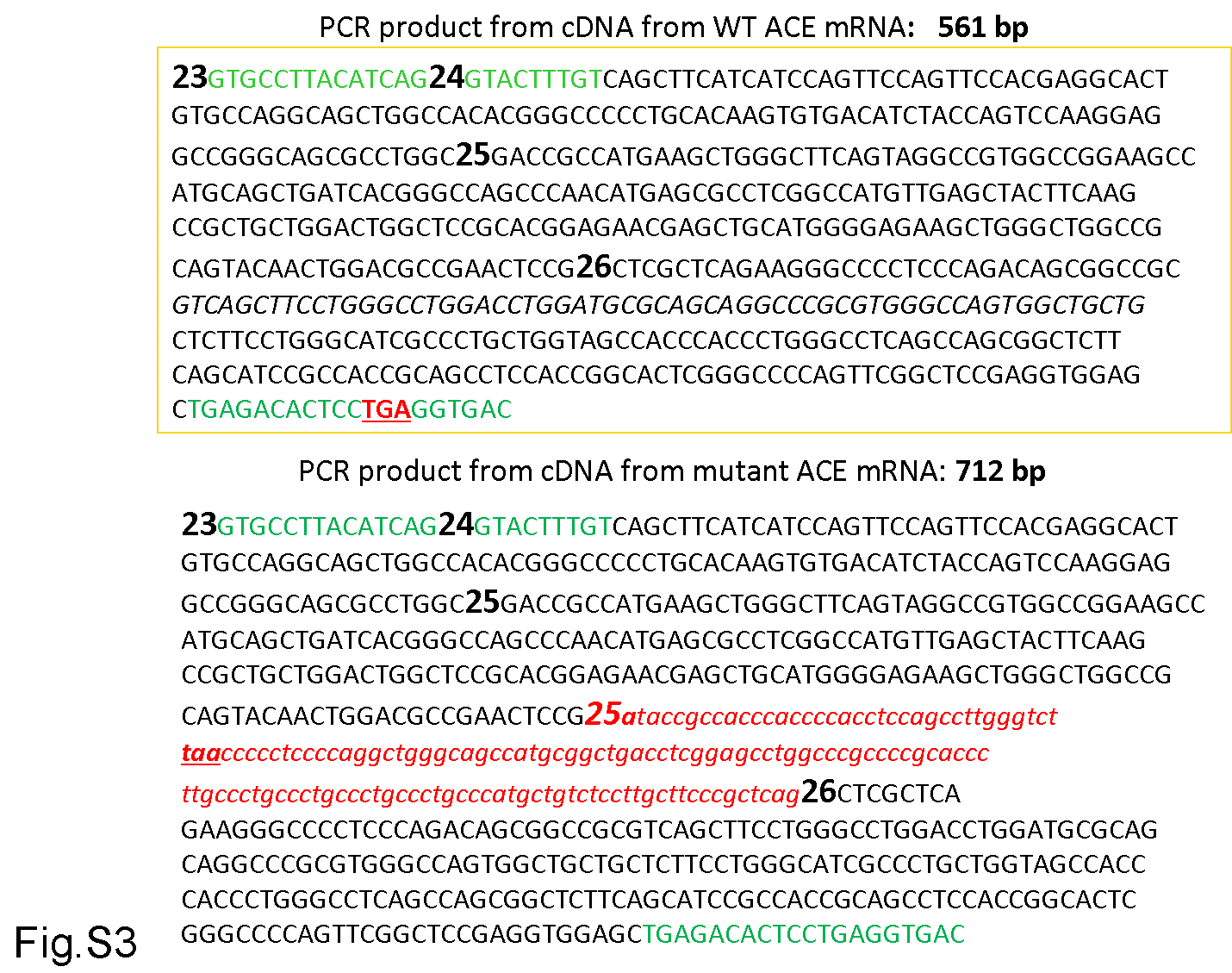

Supplement: Figure S3 — Fragment of the genomic DNA sequence. Exonic sequences (24th–26th exons) were highlighted by yellow. Substitution of 1st nucleotide in 25th intron (G) by A is shown by red color and underlined. Sequences of the primers that were used for amplification of intron-exon boundaries were highlighted with green. Amplification of cDNA (derived from mRNA) from subjects with wild type ACE (with normal level of blood ACE) gave band 561 bp. In the case of retention of 25th intron an additional band of 712 bp appeared. Putative PTC was highlighted by red. (TIF) [file pone.0059537.s003.tif]
