# Supplementary material for: A Novel Splice-Site Mutation in Angiotensin I-Converting Enzyme (ACE) Gene, c.3691+1G>A (IVS25+1G>A), Causes a Dramatic Increase in Circulating ACE through Deletion of the Transmembrane Anchor
Source: PLoS One. 2013 Apr 1;8(4):e59537. doi: 10.1371/journal.pone.0059537 (PMC3613373; doi:10.1371/journal.pone.0059537)
Supplement: Table S1 — Restriction Length Fragment Polymorphism Analysis of IVS25+1G>A mutation. (DOC) [file pone.0059537.s004.doc]

**Table** **S1**. **Restriction Length Fragment Polymorphism Analysis of IVS25+1G>A mutation. Digestion of the 292 bp fragment of WT ACE genomic DNA.**

| **Enzyme** | **Digestion site** | **1^st^ fragment** | **2^nd^ fragment** |
| --- | --- | --- | --- |
| BetI | WCCGGW | 66nt | 226nt |
| HpaII | CCGG | 67nt | 225nt |
| KpnI | GGTACC | 73nt | 219nt |
| HgiCI | GGYRCC | 69nt | 223nt |
| Acc65I | GGTACC | 69nt | 223nt |
