# Supplementary material for: A Novel Splice-Site Mutation in Angiotensin I-Converting Enzyme (ACE) Gene, c.3691+1G>A (IVS25+1G>A), Causes a Dramatic Increase in Circulating ACE through Deletion of the Transmembrane Anchor
Source: PLoS One. 2013 Apr 1;8(4):e59537. doi: 10.1371/journal.pone.0059537 (PMC3613373; doi:10.1371/journal.pone.0059537)
Supplement: Table S2 — Fold-change computation of ACE serum level according to genetic status of the patients. (DOC) [file pone.0059537.s005.doc]

**Table S2.**

Fold-change computation of ACE serum level according to genetic status of the patients.

| Variable | Parameter estimate | Std. Error | P-value | Fold-change of ACE serum level |
| --- | --- | --- | --- | --- |
| Intercept  Family  Ref: Fam 1  Fam 2  INTRON 16 (ref: I/I)  Ref: I/I  D/I  D/D  IVS 25 +1 G>A  ref : WT  MUT | 2.30  -  1.34  -  0.08  0.49  -  3.46 | 0.72  -  0.53  -  0.78  0.88  -  0.56 | 0.01  -  0.03  -  0.92  0.59  -  <0.01 | -  2.53  -  1.05  1.40  -  11.0 |
