# Supplementary material for: A Novel Splice-Site Mutation in Angiotensin I-Converting Enzyme (ACE) Gene, c.3691+1G>A (IVS25+1G>A), Causes a Dramatic Increase in Circulating ACE through Deletion of the Transmembrane Anchor
Source: PLoS One. 2013 Apr 1;8(4):e59537. doi: 10.1371/journal.pone.0059537 (PMC3613373; doi:10.1371/journal.pone.0059537)
Supplement: Data S1 — Includes in silico analysis of mutant ACE protein, RFLP identification of IVS25+1G>A ACE mutation and fold-change computation of ACE serum level according to genetic status of patients. (DOC) [file pone.0059537.s006.doc]

## Online Data Supplement

**A Novel Splice-Site Mutation in Angiotensin I-Converting Enzyme (ACE) gene, c.3691+1G>A (IVS25+1G>A), causes a dramatic increase in circulating ACE through deletion of the transmembrane anchor.**

Alexandre Persu, Michel Lambert, Jaap Deinum, Marta Cossu, Nathalie de Visscher, Leonid Irenge, Jerôme Ambroise, Jean-Marc Minon, Andrew B. Nesterovitch, Alexander Churbanov,

Isolda A. Popova, Sergei M. Danilov, A.H. Jan Danser, Jean-Luc Gala

***Analysis in silico***

Mutation affecting human splice sites can lead to two major phenotypes, exon skipping and activation of the cryptic splice sites of the same type located nearby of affected splice sites [1]. Ninety nine percents of the human internal exons have lengths less than 400nt [2], which imposes limitation on the repertoire of available cryptic splice sites. Although accurate description of the mis-spliced transcripts is critical for predicting phenotypic consequences of these alterations, their exact nature in affected individuals cannot often be determined experimentally [1].

Therefore as a first step of the analysis, two bioinformatic tools, SpliceScan II [3] and CRYP-SKIP [1], both allowing the search for potential splice sites in long-sequence stretches, were used to predict the impact of the IVS25+1G>A (c.3691+1G>A) change on splicing. Since no single prediction program will be 100% efficient in predicting the existence of likely splicing aberration, the use of more than one program is preferable [4].

According to the SpliceScan II prediction (Figure S1A), the mutation IVS25+1G>A causes the exon 25 skipping since there are no predicted 5’ splice sites located nearby. The lack of predicted exons as result of mutations means there are no gene fragments in this region that could be recognized by the spliceosome as part of mRNA. As a result of this scenario, the nucleotides from the 24^th^ exon will be attached to the nucleotides of the 26^th^ exon, that code the trans-membrane anchor in wild-type ACE. However, due to frameshift, the translated mutant protein will not contain the hydrophobic residues characteristic for the transmembrane anchor of human ACE. SRYP-SKIP prediction suggests a ~50% chance of activating two internally located 5’ splice sites in the 25^th^ exon (Figure S1B). The program predicts chances of utilization of the cryptic splice sites of the same type located nearby of normally occurring splice sites in case they are incapacitated by a mutation. Activation of these cryptic splice sites (as opposed to exon 25 skipping) would retain a fraction of the 25^th^ exon in the final mRNA product.

The observation that intron 25 is relatively short (151 nt) and flanks the terminal exon 26 is also suggestive for mutation-induced intron 25 retention where exon and intron 25 would become part of exon 26. This hypothesis is supported by observation that terminal exons are routinely longer than 600nt and are defined by molecular mechanisms different from the internal exon definition [2].

It is logical to suggest that the most likely reason for the dramatic increase in blood ACE in this family would be deletion of the transmembrane anchor, as a result of the introduction of the Premature Termination Codon (PTC) due to mutation in the canonical splice site. We found three theoretical possibilities for that.

In the 1^st^ scenario (Figure S2B), due to skipping of the 25^th^ exon, nucleotides of the 24^th^ exon will be fused to nucleotides of the 26^th^ exon. Due to frameshift, 1139 amino acid residues of human ACE will be fused with 77 non-ACE amino acid residues ending due to PTC, and this mutant ACE should have a length of 1216 amino acid residues. However, this scenario can be ruled out, because mAbs 1B8 and 5C8 bind well with ACE from subjects with IVS25+1G>A ACE mutations, and they are directed to epitopes localized in the C-terminal end of soluble ACE [5,6,7], which is coded by the 25^th^ exon.

In the next scenario, PTC will be created due to full retention of the 25^th^ intron. The sequence of the 25^th^ intron contains termination codon TAA, 36 nucleotides downstream the start of the 25^th^ intron (Figure S2C). Therefore in the case of full retention of the 25^th^ intron, 12 amino acid residues coding by the intron sequence will be attached to 1201 amino acid residues of the native human ACE, and the mutated ACE will have 1213 amino acid residues.

The third scenario would be the generation of the aberrant cryptic 5’ donor site. If such a site will be created after two nucleotides of the 25^th^ exon, the mutant mRNA will contain the original sequence from the 25^th^ exon, two nucleotides from the 25^th^ intron and 120 nucleotides downstream from the 26^th^ exon, thus leading to creation of the PTC and conversion of the hydrophobic sequence (transmembrane anchor) encoding the native 26^th^ exon into a non-hydrophobic sequence, as a result of the frameshift at the exon25/exon 26 junction (due to insertion of two nucleotides from the 25^th^ intron (Figure S2D). As a consequence, 41 non-ACE amino acid residues will be added to 1201 amino acid residues of the original human ACE, and this mutated ACE will contain 1242 amino acid residues.

***RFLP identification of IVS25+1G>A ACE mutation***.

In order to establish a simple assay for the identification of this mutation - IVS25+1G>A (c.3691+1G>A we proposed restriction fragment length (RFLP) assessment using PCR-based amplification with other pair of primers, which is based on the appearance or disappearance of the recognition sites for restrictases as a result of this mutation. The 292 bp DNA fragment was amplified using genomic DNA from carriers of this mutation in this family and individuals with normal level of ACE between exon 25 and 26 (see Materials and Methods) using genomic DNA. The five restriction endonucleases cut the 292 bp PCR product from individual with native, wild-type ACE, into two fragments as shown in Table S1. The mutation IVS25+1G>A eliminates all the restriction sites mentioned in Table S1, therefore PCR product from mutant genomic DNA should retain their length (292 bp) after digestion with these restriction endonucleases. Thus, RFLP analysis using genomic DNA can distinguish individuals with IVS25+1G>A mutations from individuals with normal ACE or carriers of other mutations in the stalk region of ACE.

The restriction fragment length polymorphism (RFLP) assessment should be performed using PCR-based amplification. The 292 bp DNA fragment should be amplified by PCR with primers Ex25_292Fw (TCCGCACGGAGAACGA, exon 25) and Ex25_292Rv (CCTGCTGCGCATCCA, exon 26) using genomic DNA from subjects with IVS25+1G>A mutation (and corresponding controls). The restriction endonucleases, mentioned in Table S1, cut the 292 bp PCR product from individual with native, wild-type ACE, into two fragments. .The mutation IVS25+1G>A eliminates all the restriction sites mentioned in Table S1.Therefore patients with this mutation will have an additional band -292 bp.

*Fold-change computation of ACE serum level according to genetic status of patients*

The fold-change of the circulating ACE level (dependent variable) produced by the IVS 25 +1 G>A mutation (independent variable) in the two pedigrees was computed using a linear regression model. Because of the potential confounding effect of the intron 16 deletion/insertion [8,9] and of the family, both variables were also included as independent variables. In the hypothesis of a multiplicative effect of these factors, the linear model was built on the log2 transformed circulating ACE levels. Through converting ACE serum values into log2 scale, the multiplicative effects were transformed into additive effects that can be estimated by the linear regression model. After parameter estimation, the fold-change caused by each variable was obtained using the inverse transformation (2^parameter^).

Compared to the baseline circulating ACE level in two I/I genotype carriers, non-significant changes in circulating ACE activity (folds-changes of 1.05 and 1.40) were observed in subjects carrying the D/I and the D/D genotypes, respectively. Compared to the wild-type genotype, the IVS 25+1 G>A mutation produced a highly significant (P-value <0.01) fold-change of 11.0.

The second family showed a significant fold-change of 2.53 in ACE levels compared to the first family. However, considering that serum ACE was measured with different biochemical methods in both families, this change does not reflect the genuine difference in ACE levels between these families.

The parameter estimates and the p-values of the linear model are given in Table S2.

**References**

1. Divina P, Kvitkovicova A, Buratti E, Vorechovsky I (2009) Ab initio prediction of mutation-induced cryptic splice-site activation and exon skipping. Eur .J. Hum. Gen. 17: 759-765.
2. Berget SM (1995) Exon Recognition in Vertebrate Splicing. J. Biol. Chem. 270: 2411-2414.
3. Churbanov A, Vorechovsky I and Hics C (2010) A method of predicting changes in human gene splicing induced by genetic variants in context of cis-acting element. BMC bioinformatics 11:22
4. Spurdle AB, Couch FJ, Hogervorst FBL, Radice P, Sinilnikova OM (2008) Prediction and assesment of splicing alterations: implications for clinical testing. Hum. Mut. 29: 1304-1313
5. Balyasnikova IV, Sun Z-L, Berestetskaya YV, Albrecht RAII, Sturrock ED, et al. (2005) Monoclonal antibodies 1B3 and 5C8 as probes for monitoring the nativity of C-terminal end of soluble angiotensin-converting enzyme (ACE). Hybridoma 24: 14-26.
6. Naperova IA, Balyasnikova IV, Schwartz DE, Watermeyer J, Sturrock ED, et al. (2008) Mapping of conformational mAb epitopes to the C domain of human angiotensin I-converting enzyme. J Proteome Res. 7:3396-3411.
7. Balyasnikova IB, Metzger R, Franke FE, Towbin H, Schwartz DE, et al. (2008) Epitope mapping of mAbs to denatured human testicular ACE. Tissue Antigens 72: 354-368.
8. Rigat B, Hubert C, Alhenc-Gelas F, Cambien F, Corvol P et al. (1990) An Insertion/Deletion Polymorphism in the Angiotensin I-converting Enzyme. Gene Accounting for Half the Variance of Serum Enzyme Levels. J. Clin. Invest. 86:1343-1346.
9. Biller H, Zissel G, Ruprecht B, Nauck M, Busse, et al. (2006) Genotype-corrected reference values for serum angiotensin-converting enzyme. Eur Respir J 28: 1085–1090.
